# ﻿*Tectariadanangensis* (Tectariaceae), a new fern species from Vietnam

**DOI:** 10.3897/phytokeys.194.80129

**Published:** 2022-04-11

**Authors:** Van The Pham, Shu-Han Li, Shi-Yong Dong

**Affiliations:** 1 Laboratory of Ecology and Environmental Management, Science and Technology Advanced Institute, Van Lang University, Ho Chi Minh City, Vietnam; 2 Faculty of Technology, Van Lang University, Ho Chi Minh City , Vietnam; 3 Key Laboratory of Plant Resources Conservation and Sustainable Utilization, South China Botanical Garden, Chinese Academy of Sciences, Guangzhou 510650, China; 4 University of Chinese Academy of Sciences, Beijing 100049, China; 5 Center of Conservation Biology, Core Botanical Gardens, Chinese Academy of Sciences, Guangzhou 510650, China

**Keywords:** Indochina, morphology, molecular phylogeny, taxonomy, *Tectariacrenata*

## Abstract

A new fern species, *Tectariadanangensis* (Tectariaceae) from Vietnam, which had long been misreported as *T.crenata*, is described and illustrated. The new species resembles *T.poilanei*, a species long neglected in the fern flora of Indochina, in the frond shape and sori arrangement, but differs by its irregularly 2-rowed sori (versus regularly 2-rowed, distantly and evenly arranged) between lateral veins of pinnae, fronds being more or less dimorphic (versus monomorphic) and basal pinnae each with a base-joined (versus free) lobe. Phylogenetic analyses of five plastid regions (*atpB*, *ndhF* + *ndhF-trnN*, *rbcL*, *rps16-matK* + *matK* and *trnL-F*) suggested *T.danangensis* has a close affinity to *T.harlandii*. *Tectariadanangensis* appears to be an intermediate species between *T.harlandii* and *T.poilanei*.

## ﻿Introduction

The fern species *Tectariacrenata* Cav. represents a morphologically distinctive group in the genus *Tectaria* Cav. (Tectariaceae). It was originally described on the basis of plants from the Mariana Islands and is characterised by the 1-pinnate fronds and indusiate sori in regular rows parallel to lateral veins of pinnae (Copeland 1907). Tardieu-Blot and Christensen (1941) recorded *T.crenata* in the flora of Indochina and cited five collections from Vietnam. However, *T.crenata* was reported by Holttum (1991) only from western Malesia, Philippines and southern Pacific Islands, but not in Indochina (including Cambodia, Laos, southern Myanmar, Thailand and Vietnam). The distribution of *T.crenata* in Vietnam remains uncertain (Hassler 2004–2021).

During recent years, we examined herbarium specimens of *Tectaria* from Asia in many herbaria and did not find any specimens of *T.crenata* from Indochina (excluding Peninsular Thailand) with typical morphology of this species as those in Malesia and Pacific Islands. The specimens from Vietnam, cited as *T.crenata* by Tardieu-Blot and Christensen (1941), turned out to represent an undescribed species which is reported here as *T.danangensis*. To test the relationships of *T.danangensis* with other species, we also conducted phylogenetic analyses of sequences of five plastid regions (*atpB*, *ndhF* + *ndhF-trnN*, *rbcL*, *rps16-matK* + *matK* and *trnL-F*).

## ﻿Methods

For morphological comparisons, we studied herbarium specimens from Indochina in Herbaria BM, BO, CDBI, E, HN, HNU, IBSC, K, KUN, L, P, PE, SING and TAIF. We also conducted field observations of *Tectaria* species in Vietnam focusing on the variations of frond dimorphism, the shape and number of lateral pinnae, venation, sori arrangement and the presence or absence of indusia.

To infer the phylogenetic position of *T.danangensis*, we assembled a sequence matrix containing five plastid regions (*atpB*, *ndhF* + *ndhF-trnN*, *rbcL*, *rps16-matK* + *matK* and *trnL-F*) of 61 specimens (Appendix 1). The sampling was based on previous phylogenetic studies of *Tectaria* by Ding et al. (2014), Zhang et al. (2017) and Dong et al. (2018). *Tectariacrenata* was revealed to be a non-monophyletic species, but its sampled specimens from western Malesia to the Solomon Islands were resolved in a strongly supported clade with *T.decurrens* (C. Presl) Copel. and *T.sulitii* Copel. (Dong et al., in press). One of the analysed specimens, *Chen et al. SITW11094* (BSIP, IBSC, TNM), was used here to represent *T.crenata*. Except for one specimen of *T.danangensis* (i.e. *Dong 4909*) which was newly sequenced and analysed in this study, other specimens were analysed in previous studies and their corresponding sequences are available in GenBank. The methods to obtain and align the five cpDNA sequences for *Dong 4909* have been as described in Ding et al. (2014).

We analysed the matrix using Bayesian Inference (BI), Maximum Likelihood (ML) and Maximum Parsimony (MP). The MP analysis was conducted in PAUP* version 4.0d100 (Swofford 2002), with all characters weighted equally and gaps treated as missing data. One thousand heuristic replicated searches were carried out using random stepwise addition with branch swapping by tree bisection-reconnection (TBR), saving 100 trees per replicate. Bootstrap values (BS) were calculated with 1000 heuristic bootstrap replicates, one random sequence addition and TBR swapping. For BI and ML analyses, we used the software jModelTest (Posada 2008) to determine the best-fitting substitution models for the concatenated sequences and the results suggested GTR+G+I as the best-fitting model. The BI analysis was conducted with MrBayes 3.2.6 (Ronquist et al. 2012), using 10 million generations with one tree sampled every 1,000 generations; four runs with four chains were performed in parallel. The first 25% trees were discarded as burn-in. The ML analysis was conducted using raxmlGUI 2.0 (Edler et al. 2020). A thorough tree search for the best ML tree was performed. The ML bootstrap analysis was performed with 1000 replications. The analysed sequence matrix and resulting trees are available in Dryad Digital Repository (https://doi.org/10.5061/dryad.51c59zw9t).

## ﻿Results

**Figure 1.**
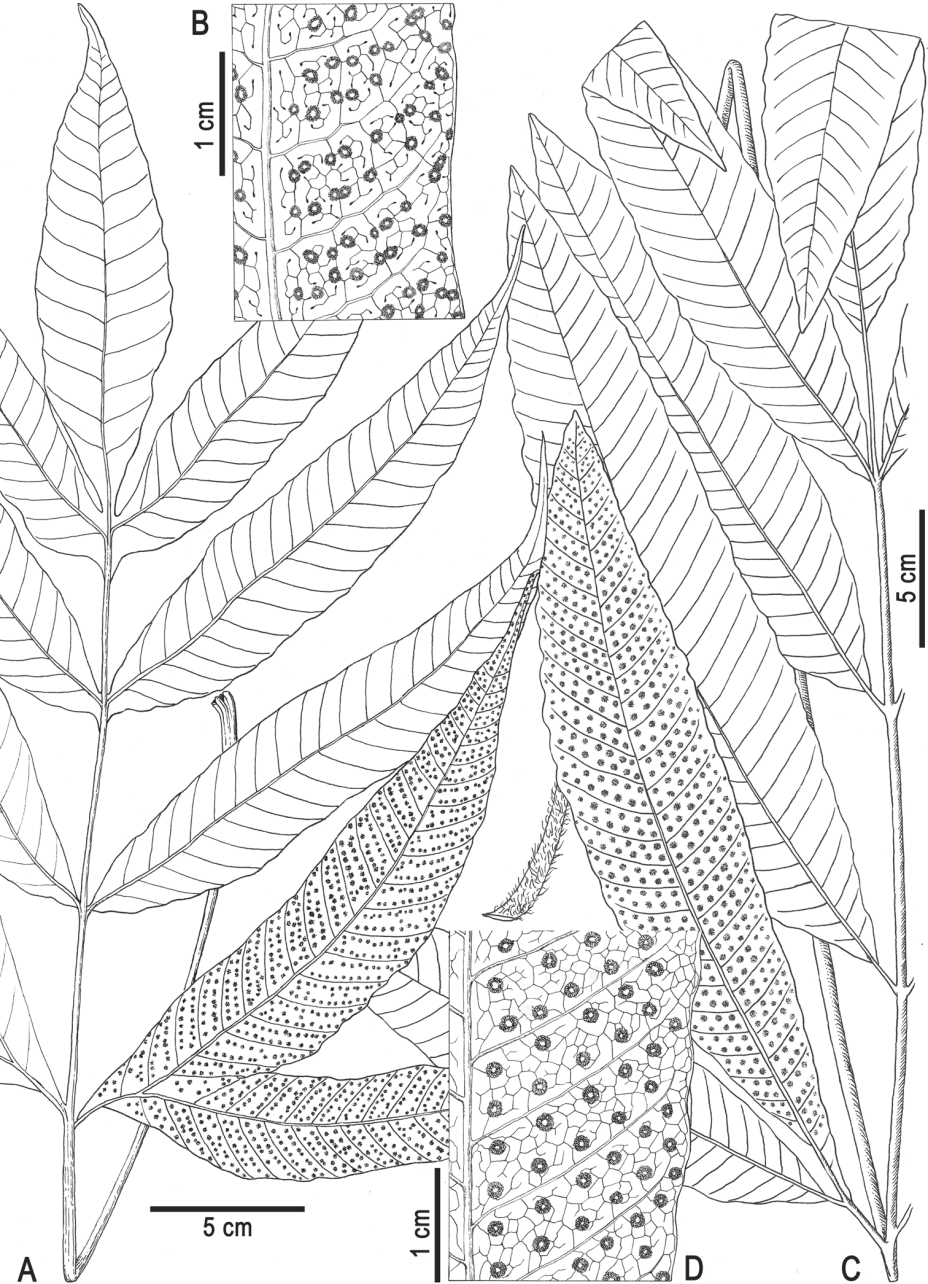
Morphological comparison between *Tectariadanangensis* (**A, B**) and *T.poilanei* (**C, D**) **A, C** habit **B, D** detail of a pinna showing venation and sori arrangement. Drawn by Shu-Han Li, with **A** and **B** based on *Dong 4909* (holotype, IBSC) and **C** and **D** on *Poilane 24074* (holotype, P).

**Figure 2.**
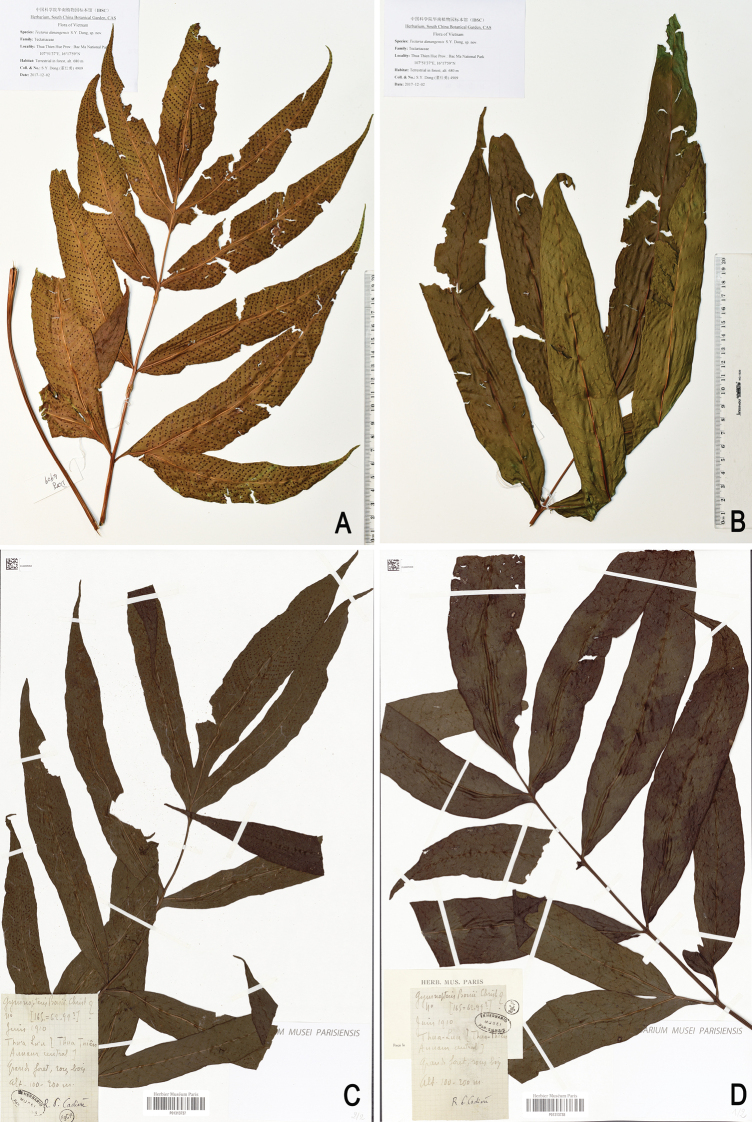
Herbarium specimens of *Tectariadanangensis*, showing contracted fertile fronds (**A, C**) as compared with sterile fronds (**B, D**) **A, B***Dong 4909* (type, IBSC) **C, D***Cadiere 165* (P).

Morphological comparisons showed that the specimens recorded as *T.crenata* by Tardieu-Blot and Christensen (1941) represent an undescribed species which is recognised as *T.danangensis*. This new species superficially resembles *T.crenata* in the 1-pinnate fronds and entire pinnae, but distinctly differs in sori features, such as being borne on anastomosing veins (versus terminal on free veins included in areoles) and in irregular two rows (versus regularly 2-rowed, distantly and evenly arranged) between lateral veins of pinnae/segments (Fig. 1A and B). Based on herbarium specimens and recent collections, we found that *T.danangensis* is quite variable in the frond dimorphism, with fertile fronds contracted to different extents compared with sterile ones and its sori are in irregular two rows between lateral veins, close or distant to each other. A few specimens of *T.danangensis* with less contracted fertile fronds are similar to those of *T.poilanei* Tardieu, but differ mainly in their irregular 2-rowed sori (versus regularly 2-rowed) between lateral veins, upper pinnae mostly being adnate (versus pointed) to rachis and basal pinnae each having a basiscopic base-joined (versus free) lobe (Figs 1 and 2). We detected a total of 25 herbarium collections of *T.danangensis* containing fertile fronds, of which seven collections bore evidently abortive sporangia.

**Figure 3.**
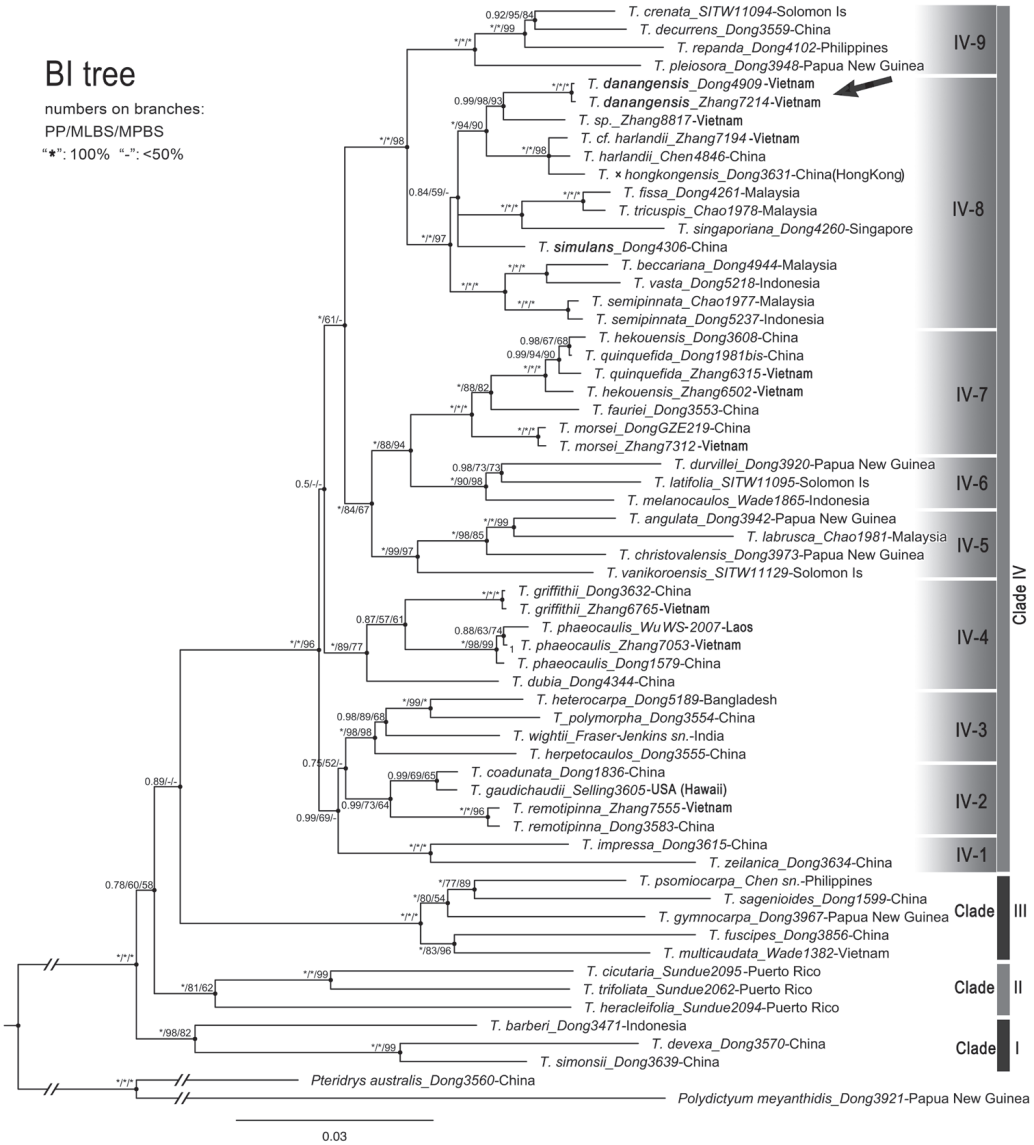
Bayesian consensus tree of *Tectaria*, based on combined plastid regions of *atpB*, *ndhF + ndhF-trnN*, *rbcL*, *rps16-matK + matK* and *trnL-F*. The position of the new species, *T.danangensis*, is indicated by an arrow.

Our phylogenetic analyses of cpDNA sequences with all three methods (BI, ML or MP) consistently resolved *T.danangensis* in Clade IV-8 of *Tectaria* (Fig. 3). Based on the current sampling, two specimens of *T.danangensis* and an unidentified specimen (*Zhang et al. 8817*, for which we had no chance to examine the morphology) formed a strongly support sister relationship with *T.harlandii* clade including T.×hongkongensis S.Y. Dong and a *T.harlandiii*-like specimen (PP = 1.0, MLBS = 94% and MPBL = 90%). In contrast, *T.crenata* and allied species were resolved in a different clade (IV-9, Fig. 3). Though *T.danangensis* was suggested as having a close affinity to *T.harlandii* (Hook.) C.M. Kuo, these two species are morphologically strikingly different in sori features. Specifically, *T.danangensis* has discrete sori, whereas *T.harlandii* has nearly acrostichoid sori. A comparison of morphological characters amongst *T.danangensis*, *T.poilanei* and *T.harlandii* is listed in Table 1.

**Table 1.** Morphological differences amongst *T.danangensis*, *T.fissa*, *T.harlandii* and *T.poilanei*.

	*T.harlandii*	*T.danangensis*	*T.poilanei*	*T.fissa*
Frond dimorphism	Strongly dimorphic	Mostly semi-dimorphic	Monomorphic	Monomorphic
Number of lateral pinnae/segments	1–3 pairs	2–5(6) pairs	3–4 pairs	1–5 pairs
Upper pinnae/segments	Adnate to rachis, mostly connate with terminal segment at base	Adnate to rachis, connate with terminal segment or not	Free, shortly petiolulate or sessile	Adnate to rachis, connate with terminal segment or not
Lobes on basal pinnae	Absent	Present; their bases connate to basal pinnae	Present; their bases cuneate, sessile or shortly petiolulate	Mostly present; their bases cuneate or connate
Wingless petioles of basal pinnae	Absent	Almost absent, 0–0.5 cm long	1.2–2 cm long	0–1.5 cm long
Transverse veins between lateral veins of pinnae	Distinct on sterile fronds, absent on fertile fronds	Variable, mostly indistinct	Absent	Distinct
Sori	Nearly acrostichoid, with sporangia running along veins between lateral veins	Round; irregularly in 2 rows between lateral veins, close or distant	Round; regularly in 2 rows between lateral veins, uniformly distant	Round; irregularly in 4–6 rows between lateral veins, close to each other
Indusia	Absent	Present	Present	Present

## ﻿Discussion

Tardieu-Blot and Christensen (1941) overlooked the sori differences between *T.danangensis* and *T.crenata* and misidentified the former as the latter in Vietnam. Though having similar shape and dissection of fronds to *T.danangensis*, *T.crenata* and its allied species in Clade IV-9 (Fig. 3) (including *T.decurrens*, *T.pleiosora* (Alderw.) C. Chr. and *T.repanda* (Willd.) Holttum) differ from *T.danangensis* in their characteristic sori which are large and regularly 2-rowed between lateral veins, with each sorus being terminal on a single veinlet in an areole (Tagawa and Iwatsuki 1988: 372; Holttum 1991: 80). Such sori features are stable in these species and can be considered as a synapomorphy for Clade IV-9. In contrast, for species in Clade IV-8, the sori are never in regular two rows between lateral veins nor terminal on free veinlets included in areoles. Instead, their sori are relatively small, scattered between lateral veins and mostly borne on anastomosing veins in most species clustered in Clade IV-8, except for *T.danangensis*, *T.harlandii* and T.×hongkongensis. *Tectariadanangensis* has a unique arrangement of sori which are in irregular two rows between lateral veins (Fig. 1A and B); while in *T.harlandii* and T.×hongkongensis, the sori are nearly acrostichoid, with sporangia running along veinlets between lateral veins, as shown in Zhao and Dong (2016: Fig. 2C).

By examining specimens of all *Tectaria* species with 1-pinnate, pinnae-entire fronds recorded in Indochina and nearby regions (Tardieu-Blot and Christensen 1941; Tagawa and Iwatsuki 1988; Xing et al. 2013; Fraser-Jenkins et al. 2018), we found that some specimens of *T.danangensis* look very like those of *T.fissa* (Kunze) Holttum, a species frequently occurring in western Malesia but not in Indochina (Holttum 1991; Lindsay and Middleton 2012 onwards). A detailed comparison (Table 1) showed that *T.danangensis* differs from *T.fissa* and other species having 1-pinnate fronds by its venation lacking distinct transverse veins between lateral veins and its sori being generally in only two rows (versus 4–6 rows) between lateral veins.

*Tectariadanangensis* appears to be an intermediate species between *T.harlandii* and *T.poilanei*; the latter (*T.poilanei*) has long been neglected in literature accounting for the fern flora of Indochina (e.g. Tardieu-Blot and Christensen 1941; Tagawa and Iwatsuki 1988; Phan 2010; Lindsay and Middleton 2012 onwards). According to herbarium specimens examined, *T.danangensis* is not rare in Vietnam; it has been collected from 1837 to 2014 across nearly all the country and, morphologically, is quite variable in the frond dimorphism and sori distribution between lateral veins. As shown in Table 1, some characters in *T.danangensis*, such as frond dimorphism, attachment pattern of pinnae to rachis, venation and sori distribution, exhibit intermediate states of those between *T.harlandii* and *T.poilanei*. Notably, *T.poilanei* is quite stable in pinnae features (i.e. the broad-lanceolate shape, lower pinnae consistently being petiolulate and basal pinnae each bearing a free basiscopic lobe), venation lacking transverse veins between lateral veins and regularly 2-rowed well-spaced sori (Fig. 1C and D); this species is currently represented, so far as we know, by its type specimen from southern Vietnam (Tardieu-Blot 1940) and a few collections from Thailand extend its distribution (e.g. *Beusekom & Smitinand 2193* (L) from Chantaburi, *Hansen & Smitinand 12644* (K, L) from Mae Hong Son and *Maxwell 04-156* (L) and *Hansen et al*. *10886* (K, L) from Chiang Mai). Based on its variable morphology and frequently abortive sporangia, we hypothesised that *T.danangensis* possibly involved hybridisation with other species. Further studies, especially chromosome number and reproductive mode, are needed to better determine the origin of *T.danangensis* and its relationships with other *Tectaria* species.

## ﻿Taxonomic treatment

### ﻿ Tectaria danangensis

Taxon classificationPlantaePolypodialesTectariaceae

S.Y. Dong sp. nov.

FD5ABEF6-C8A6-5646-9E96-2A58FDE4D2E1

urn:lsid:ipni.org:names:77296979-1

Figs 1A, B , 2

#### Type.

**Vietnam.** On the border between Da Nang and Thua Thien Hue Prov.: Bach Ma National Park, 107°51'37"E, 16°17'59"N, 680 m elev., 02 Dec 2017, *S.Y. Dong 4909* (holotype: IBSC!, designated here; isotypes: HNU!, IBSC!).

#### Diagnosis.

*Tectariadanangensis* is similar to *T.poilanei* Tardieu, but differs in its irregularly 2-rowed sori (versus regularly 2-rowed, well-spaced and evenly arranged) between lateral veins, fronds more or less being dimorphic (versus monomorphic) and basal pinnae each having a base-joined (versus free) lobe.

#### Description.

**Rhizome** short, erect or decumbent. **Fronds** more or less dimorphic, with fertile fronds slightly contracted; stipe reddish-brown, 3–4 mm in diameter, 30–50 cm long, bearing scales only at base; scales lanceolate, ca. 8–10 × 1–1.5 mm, reddish-brown; lamina nearly round or oblong, 35–55 × 25–35 cm, imparipinnate or terminated by tri-lobed segments, having 2–4(6) pairs of lateral pinnae, pinnae and segments entire at margin, herbaceous in texture, hairless; basal pinnae forked, 14–27(33) × 1.7–5 cm, petiolules 0–5 mm long, acroscopic base cuneate, basiscopic base round, apex caudate-acuminate, having a basiscopic lobe, the basiscopic lobes 7–24 × 1–3.5 cm; suprabasal pinnae linear, 14–30 × 1–4.5 cm, sessile, base cuneate, apex acuminate or caudate; upper pinnae similar to suprabasal pinnae in size and shape, but mostly adnate to rachis. **Veins** fully anastomosing, with most areoles having included free or forked veinlets, transverse veins between lateral veins mostly indistinct. **Sori** round, borne on anastomosing veins, generally in two rows between lateral veins of pinnae (more or less with additional sori present beyond two rows), 8–11 each row in broad pinnae or 4–6 in obviously contracted fertile pinnae, well-spaced or adjacent. **Indusia** round-reniform, mostly curled and almost covered by sporangia when mature.

#### Distribution and habitat.

Vietnam (Da Nang, Lam Dong, Quang Binh, Quang Nam, Quang Tri, Thanh Hoa, and Thua Thien Hue); terrestrial in broadleaved evergreen forest, occurring in slopes of valley or along mountain ridge, elev. 200–1400 m, locally common.

#### Additional specimens examined

**(paratypes).**

**Vietnam. Da Nang**: Ba Na Mountain, Hoa Vang District, *Sallet s.n.* (P); without locality, *Gaudichaud s.n.* (P). **Lam Dong**: Da Lat, *Wu et al*. *WP1447* (HN). **Quang Binh**: Phong Nha – Ke Bang National Park, *Nguyen NT39*, *NT69 & NT102* (HNU); without locality, *Phan s.n.* (HNU). **Quang Nam**: without locality, *Poilane 29484 & 31661* (P). **Quang Tri**: Huong Hoa District, *Averyanov et al*. *CPC2906 & CPC2907* (HNU); Dakrong District, *Phan et al*. *HLF6122* (HNU); Dakrong Nature Reserve, *Lu 19232* (TAIF); “Mai-lanh”, *Poilane 1189* (P, PE, SING). **Thanh Hoa**: Phu luc, *Lecomte & Finet 1338* (P). **Thua Thien Hue**: A Luoi District, *Averyanov et al*. *HAL7289*, *HAL7342*, *HAL7423*, *HAL7622*, *HAL7738* (HNU); Nam Dong District, *Averyanov et al*. *HAL6940* (HNU); “Tua Luu”, *Cadiere 165* (P). **Southern Vietnam** (Annam, with localities’ names unreadable): *Eberhardt 373* (P).

## Supplementary Material

XML Treatment for Tectaria danangensis

## ﻿Appendix 1

Accessions used for phylogenetic analyses in this study. Information are arranged in this order: species name, voucher specimen (collector, number, and herbarium), place of origin, and GenBank numbers for *rbcL*, *atpB*, *rps16-matK* + *matK*, *ndhF* + *ndhF-trnN*, and *trnL-F* spacer (NA indicates data absent).

***Polydictyummenyanthidis*** (C.Presl) Copel., *Dong 3921* (IBSC, LAE), Papua New Guinea (Lae), MF623752/MF623680/MF623704/MF623728/MF623776. ***Pteridrysaustralis*** Ching, *Dong 3560* (IBSC), China (Yunnan), KJ196892/KJ196486/KJ196796/KJ196678/KJ196522. ***Tectariaangulata*** (Willd.) Copel., *Dong 3942* (IBSC, LAE), Papua New Guinea (Kimbe), MF623756/MF623756/MF623708/MF623732/MF623779. ***Tectariabarberi*** (Hook.) Copel., *Dong 3471* (IBSC), Indonesia (Kalimantan), KJ196846/KJ196445/KJ196584/KJ196778/KJ196628. ***Tectariabeccariana*** (Ces.) C.Chr., *Dong 4944* (IBSC), Indonesia (Java), OK104174/OK480073/OK480115/OK480153/OK480194. **Tectariacf.harlandii** (Hook.) C.M.Kuo, *Zhang et al*. *7194* (CDBI), Vietnam (Ha Tinh), NA/NA/KY937237/NA/KY937513. ***Tectariachristovalensis*** (C.Chr.) Alston, *Dong 3973* (IBSC), Papua New Guinea (Kimbe), OK104179/OK480081/OK480121/OK480161/OK480202. ***Tectariacicutaria*** (L.) Copel., *Sundue 2095* (VT), Puerto Rico, KJ196905/KJ196408/KJ196621/KJ196729/KJ196696. ***Tectariacoadunata*** (J.Sm.) C.Chr., *Dong 1836* (IBSC), China (Yunnan), KJ196878/KJ196451/KJ196531/KJ196779/KJ196661. ***Tectariacrenata*** Cav., *Chen et al*. *SITW11094* (BSIP, IBSC, TNM), Solomon Islands (Temotu), OK104204/OK480084/OK480124/OK480164/OK480205. ***Tectariadanangensis*** S.Y.Dong, *Dong4909* (HNU, IBSC), Vietnam (Da Nang), OM671282/OM671283/OM671284/OM671285/OM671286; *Zhang et al*. *7214* (CDBI), Vietnam (Quang Binh), KY937368/NA/KY937261/NA/KY937550. ***Tectariadecurrens*** (C.Presl) Copel., *Dong 3559* (IBSC), China (Yunnan), KJ196870/KJ196471/KJ196535/KJ196741/KJ196674. ***Tectariadevexa*** (Kunze) Copel., *Dong 3570* (IBSC), China (Yunnan), KJ196883/KJ196456/KJ196787/KJ196668/KJ196605. ***Tectariadubia*** (C.B.Clarke & Baker) Ching, *Dong 4344* (IBSC), China (Yunnan), MF623762/MF623690/MF623714/MF623738/MF623783. ***Tectariadurvillei*** (Bory) Holttum, *Dong 3920* (IBSC, LAE), Papua New Guinea (Lae), MF623763/MF623691/MF623715/MF623739/MF623784. ***Tectariafauriei*** Tagawa, *Dong 3553* (IBSC), China (Yunnan), KJ196887/KJ196480/KJ196526/KJ196792/KJ196671. ***Tectariafissa*** (Kunze) Holttum, *Dong 4261* (IBSC), Malaysia (Selangor), MF623766/MF623694/MF623718/MF623742/MF623787. ***Tectariafuscipes*** (Bedd.) C.Chr., *Dong 3856* (IBSC), China (Hainan), MF623767/MF623695/MF623719/MF623743/MF623788. ***Tectariagaudichaudii*** Maxon, *Selling 3605* (H), USA (Hawaii), KF887176/NA/NA/NA/NA. ***Tectariagriffithii*** (Baker) C.Chr., *Dong 3632* (IBSC), China (Guangxi), KJ196872/KJ196473/KJ196578/KJ196775/KJ196651; *Zhang et al*. *6765* (CDBI), Vietnam (Bac Kan), KY937337/NA/KY937229/NA/KY937501. ***Tectariagymnocarpa*** Copel., *Dong 3967* (IBSC), Papua New Guinea (Kimbe), MF623765/MF623693/MF623717/MF623741/MF623786. ***Tectariaharlandii*** (Hook.) C.M.Kuo, *Chen et al*. *4846* (IBSC), China (Guangdong), KJ196839/KJ196432/KJ196612/KJ196758/KJ196718. ***Tectariahekouensis*** Ching & Chu H.Wang, *Dong 3608* (IBSC), China (Yunnan), KJ196894/KJ196487/KJ196520/KJ196799/NA; *Zhang et al*. *6502* (CDBI), Vietnam (Hanoi), KY937340/NA/KY937238/NA/KY937514. ***Tectariaheracleifolia*** (Willd.) Underw., *Sundue 2094* (VT), Puerto Rico, KJ196904/KJ196407/KJ196597/KJ196728/KJ196695. ***Tectariaherpetocaulos*** Holttum, *Dong 3555* (IBSC), China (Yunnan), KJ196884/KJ196482/KJ196570/KJ196789/KJ196669. ***Tectariaheterocarpa*** (Bedd.) C.V.Morton, *Dong 5189* (IBSC), Bangladesh (Sylhet), MW795598/MW795606/MW795612/MW795620/MW795628. ***Tectaria×hongkongensis*** S.Y.Dong, *Dong 3631* (IBSC), China (Hong Kong), KJ196886/KJ196484/KJ196568/KJ196783/KJ196666. ***Tectariaimpressa*** (Fée) Holttum, *Dong 3615* (IBSC), China (Yunnan), KJ196841/KJ196420/KJ196536/KJ196772/KJ196626. ***Tectarialabrusca*** (Hook.) Copel., *Chao 1981* (TAIF), Malaysia (Sarawak), KJ196818/KJ196499/KJ196600/KJ196745/KJ196692. ***Tectarialatifolia*** (G.Forst.) Copel., *Chen et al*. *SITW11095* (BSIP, IBSC, TAIF, TNM), Solomon Islands (Temotu), OK104190/OK480096/OK480135/OK480174/OK480217. ***Tectariamelanocaulos*** (Blume) Copel., *Chen Wade1865* (TAIF), Indonesia (Java), KJ196832/KJ196422/KJ196562/KJ196735/KJ196709. ***Tectariamorsei*** (Baker) S.Y.Dong, *Dong GZE219* (IBSC), China (Guizhou), KJ196893/KJ196418/KJ196521/KJ196798/KF561675; *Zhang et al*. *7312* (CDBI), Vietnam (Quang Binh), KU605205/NA/KU605139/NA/KU605117. ***Tectariamulticaudata*** (C.B.Clarke) Ching, *Chen Wade1382* (TAIF), Vietnam (Bu Gia Map National Park), KJ196834/KJ196425/KJ196558/KJ196756/KJ196713. ***Tectariaphaeocaulis*** (Rosenst.) C.Chr., *Dong 1579* (IBSC), China (Hainan), KJ196879/KJ196453/KJ196546/KJ196780/KJ196662; *Zhang et al*. *7053* (CDBI), Vietnam (Thanh Hoa), KU605201/NA/KU605142/NA/KU605119; *Wu WS*-*2007* (KUN, MO), Laos, NA/NA/NA/NA/KY937532. ***Tectariapleiosora*** (Alderw.) C.Chr., *Dong 3948* (IBSC, LAE), Papua New Guinea (Kimbe), MF623759/MF623687/MF623711/MF623735/MF623793. ***Tectariapolymorpha*** (Hook.) Copel., *Dong 3554* (IBSC), China (Yunnan), J196889/KJ196477/KJ196524/KJ196794/KJ196657. ***Tectariapsomiocarpa*** S.Y.Dong, *Chen s.n.* (TAIF), Philippines (Luzon), J196822/KJ196502/KJ196595/KJ196723/KJ196698. ***Tectariaquinquefida*** (Baker) Ching, *Dong 1981bis* (IBSC), China (Yunnan), KJ196885/KJ196483/KJ196528/KJ196890/KJ396622; *Zhang et al*. *6315* (CDBI), Vietnam (Hoa Binh), KY937358/NA/KY937250/NA/KY937537. ***Tectariaremotipinna*** Ching & Chu H.Wang, *Dong 3583* (IBSC), China (Yunnan), KJ196851/KJ196450/KJ196574/KJ196781/KJ196663; *Zhang et al*. *7555* (CDBI), Vietnam (Quang Tri), KY937325/NA/NA/NA/KY937482. ***Tectariarepanda*** (Willd.) Holttum, *Dong 4102* (IBSC), Philippines (Palawan), OK104195/OK480104/OK480144/OK480183/OK480228. ***Tectariasagenioides*** (Mett.) Christenh., *Dong 1599* (IBSC), China (Hainan), KJ196896/KJ196436/KJ196550/KJ196760/KJ196625. ***Tectariasemipinnata*** (Roxb.) C.V.Morton, *Chao 1977* (TAIF), Malaysia, KJ196817/KJ196498/KJ196601/KJ196744/KJ196691; *Dong 5237* (IBSC), Indonesia (Sumatra), NA/OK480106/OK480146/OK480185/OK480230. ***Tectariasimonsii*** (Baker) Ching, *Dong 3639* (IBSC), China (Guangxi), KJ196837/KJ196430/KJ196555/KJ196730/KJ196717. ***Tectariasimulans*** Ching, *Dong 4306* (IBSC), China (Yunnan), OK104197/OK480108/OK480148/OK480187/OK480231. ***Tectariasingaporiana*** (Hook. & Grev.) Copel., *Dong 4260* (IBSC), Singapore, MF623771/MF623699/MF623723/MF623747/MF623791. ***Tectaria* sp.**, *Zhang et al*. *8817* (CDBI), Vietnam (Khanh Hoa), NA/NA/KY937269/NA/KY937557. ***Tectariatricuspis*** (Bedd.) Copel., *Chao 1978* (TAIF), Malaysia (Kuala Lumpur), KJ196820/KJ196501/KJ196598/KJ196847/KJ196694. ***Tectariatrifoliata*** (L.) Cav., *Sundue 2062* (VT), Puerto Rico, KJ196901/KJ196409/KJ196565/KJ196848/NA. ***Tectariavanikoroensis*** S.Y.Dong & C.W.Chen (ined.), *Chen et al*. *SITW11129* (BSIP, IBSC, TNM), Solomon Islands (Temotu), OK104200/OK480111/NA/OK480190/OK480234. ***Tectariavasta*** (Blume) Copel., *Dong 5218* (IBSC), Indonesia (Sumatra), NA/OK480112/OK480151/OK480191/NA. ***Tectariawightii*** (C.B.Clarke) Ching, *Fraser-Jenkins s.n.* (TAIF), India (Kerala), KJ196906/KJ196416/KJ196561/KJ196732/KJ196710. ***Tectariazeilanica*** (Houtt.) Sledge, *Dong 3634* (IBSC), China (Yunnan), KJ196862/KJ196442/KJ196540/KJ196768/KJ196637.
